# Homocysteine Mediates Cognitive Inflexibility Induced by Stress via Targeting PIN1

**DOI:** 10.3390/brainsci15040416

**Published:** 2025-04-19

**Authors:** Xiaobing Chen, Ling Zhang, Bing Liu, Huafeng Dong, Shijia Zhang, Xue Wang, Zhaowei Sun, Fang Xie, Lingjia Qian, Yun Zhao

**Affiliations:** Beijing Institute of Basic Medical Sciences, Beijing 100039, China; 17859518569@163.com (X.C.); 18306826208@163.com (L.Z.); liubingzsp@163.com (B.L.); feng13191908265@163.com (H.D.); zsjdeyx2022@163.com (S.Z.); snowwang0326@foxmail.com (X.W.); sunzhw0820@163.com (Z.S.); vancoxie1@163.com (F.X.); stressqian@163.com (L.Q.)

**Keywords:** cognitive flexibility, HCY, PIN1, stress

## Abstract

Background: Increasing evidence shows that HCY plays an important role in stress-induced cognitive dysfunction, and HCY significantly promotes the decline of cognitive function. Stress has been reported to cause elevated HCY in the hippocampus of mice. Cognitive flexibility refers to the ability of individuals to quickly adjust their neurobehavioral strategies to different situations or to solve different tasks. Aims: This study aims to explore the role of HCY in the impairment of cognitive flexibility induced by stress and its possible regulatory mechanism. Methods and Results: First, we examined changes in the protein and mRNA levels of the cognitive flexibility effector molecule, PIN1, during stress in mice. The results show that stress can cause a decline in cognitive flexibility in mice and lead to an increase in PIN1. Moreover, through the use of in vitro experiments, we found that HCY could induce an increase in PIN1 expression in neurons. Further in vivo experiments were used to investigate the effect of VitB on HCY and PIN1 and evaluated the therapeutic effect of VitB on stress-induced impairment of cognitive flexibility. The results show that VitB decreased the levels of HCY in plasma and the hippocampus, alleviated the stress-induced impairment of cognitive flexibility, and reduced the expression of PIN1. Conclusions: These results suggest that the impairment of cognitive flexibility induced by stress can be inhibited by regulating the content of HCY. Collectively, our findings highlight therapeutic strategies aimed at improving HCY treatment for impairments in cognitive flexibility.

## 1. Introduction

Cognitive flexibility refers to the ability of individuals to quickly adjust their neurobehavioral strategies to different situations or to solve different tasks. Cognitive flexibility is an important part of executive function, which belongs to the advanced function of cognitive ability. It involves the rapid processing of information, the switching of thinking, and the ability to adapt to new situations [1,2]. Clinical studies have shown that many neuropsychiatric diseases cause cognitive flexibility impairment [3]. The underlying mechanisms of impaired cognitive flexibility involve multiple complex biological processes, including aberrant activation of the hypothalamic–pituitary–adrenal axis, dysregulation of the neurotransmitter system, and metabolic abnormalities [4,5,6], but the specific mechanism of cognitive flexibility impairment is not clear. Long-term exposure to stressful environments can trigger a series of physiological and psychological responses, among which impairment of cognitive flexibility is a common response [7,8]. Studies have shown that chronic restraint stress is closely related to the occurrence and development of cognitive dysfunction, which is an important feature of cognitive flexibility decline [9,10]. Accumulating evidence suggests that chronic stress, including Chronic Unpredictable Mild Stress (CUMS), social isolation, and restraint stress, can reduce hippocampal neurogenesis in adolescents, leading to impaired hippocampus-dependent learning and memory and depression-like behaviors [11]. In addition, the hippocampus is involved in memory updating, spatial processing, and neuroplasticity, all of which underlie cognitive flexibility [12,13,14]. Therefore, it is of great significance to study how stress promotes the decline of cognitive flexibility, reveal the pathophysiological mechanisms of cognitive flexibility impairment, and develop effective therapeutic interventions. Mice have been reported to have impaired cognitive flexibility, so in this study, we used mice as model animals for the experimental studies [15].

Peptidyl-prolyl cis-trans isomerase NIMA-interacting 1(PIN1) is a proline isomerase and is capable of regulating the activity of the NIMA-interacting enzyme; it affects protein folding, stability, function, and the interaction between other proteins [16]. PIN1 is widely expressed in the central and peripheral nervous system regions and is involved in the regulation of neuronal development, apoptosis, and synaptic activity; it plays an important role in many neurodegenerative disorders, such as Alzheimer’s disease (AD) and Parkinson’s disease (PD). Recent studies have found a potential link between PIN1 and cognitive flexibility [17,18,19]. Chronic stress can affect cognitive function by activating the microglia and triggering neuroinflammation. PIN1 may play a role in the stress response and neuroinflammation by regulating the signaling pathways and protein functions of neurons, thus affecting cognitive flexibility [20,21]. Therefore, modulating the expression of PIN1 may be a potential therapeutic strategy for improving cognitive impairment and related neurological disorders.

Homocysteine (HCY) is a sulfur-containing amino acid that is an important intermediate in the metabolism of methionine and l-cysteine. The metabolism of HCY involves two main pathways: remethylation and transsulfurization. Vitamin B12, Vitamin B6, and folic acid are key cofactors in these metabolic processes. Nutritional deficiency, genetic factors, drug interference, or renal dysfunction may lead to abnormal metabolism of HCY, and may then cause HCY accumulation in the body. Supplementation with VitB (including VitB_6_, VitB_12_, folic acid) is an effective way to reduce HCY levels [10,22,23]. Elevated HCY levels are an independent risk factor for a variety of diseases, these include cerebrovascular disease (arteriosclerosis, stroke, coronary artery disease), neurodegeneration (AD, PD), complications of diabetes, osteoporosis, chronic kidney disease, and male infertility [24,25,26,27,28]. More and more studies have shown that HCY plays a key role in the pathogenesis of stress-related diseases [10,29,30]. Previous studies in the laboratory have found that chronic restraint stress can cause abnormally high HCY and abnormal metabolic pathways [10], and HCY metabolic abnormalities are involved in cognitive and emotional disorders caused by stress; however, whether HCY mediates stress-induced impairment of cognitive flexibility by targeting PIN1 is not fully understood. Available evidence indicates that chronic restraint stress can lead to increased HCY levels in the plasma and hippocampus of mice [10]. In addition, HCY can damage the blood–brain barrier (BBB), increasing vascular permeability, which in turn causes peripheral inflammatory factors to enter the brain, further exacerbating cognitive impairment [31,32]. Therefore, regulating HCY levels may be an important strategy to alleviate cognitive impairment induced by restraint stress.

In this study, our main focus was to elucidate the role and possible targets of HCY in regard to restraint stress-induced impairment of cognitive flexibility. Our study found that restraint stress can cause impairment of cognitive flexibility, as a result of an increase in the expression of PIN1 and abnormally elevated HCY levels. With the downregulation of the HCY level, the impairment of cognitive flexibility was alleviated, and HCY was negatively correlated with cognitive flexibility. Meanwhile, the expression of PIN1 was inhibited.

## 2. Materials and Methods

### 2.1. Animal Experiment

Male wild-type C57BL/6 mice (8 weeks) were obtained from HuaFuKang Company (Beijing, China) and randomly allocated to control and experimental groups. All the animal procedures were approved by the Institutional Animal Care and Use Committee at the Academy of Military Medical Sciences and were performed in accordance with the National Research Council’s Guide for the Care and Use of Laboratory Animals (8th edition). Following the completion of the behavioral experiments, the mice were anesthetized via the intraperitoneal injection of Tribromoethanol (Meilunbio, MA0478, Dalian, China) at a dose of 30 µL/g of body weight.

### 2.2. Restraint Stress Procedure

After 1 week of adaptation, the mice were subjected to restraint stress, according to the experimental procedure. The restraint stress (RS) applied in this study followed the methods described in previous experiments [33]. In short, this involved placing the mice head down, alone, in a well-ventilated 50 mL polypropylene cone tube and then inserting them into a 3 cm long intermediate tube; finally, the lid of the 50 mL tube was fastened, so that the mice could not rotate freely inside the restraint tube. Then, the restraint tube containing the mice was placed into a plastic cage, with the head end elevated; after restraint, the mice were placed back into the plastic cages where they had been housed previously and were given free access to food and water. In this study, the restraint procedure lasted from 22:00 p.m. to 8:00 a.m.

### 2.3. Morris Water Maze (MWM)

The mice were moved from the animal room to the Behavioral Laboratory 1 h before the experiment began. The mice were subjected to two consecutive MWM tests: the spatial water maze (sMWM) and the inverted water maze (rMWM). The spatial water maze assessed spatial learning and memory in mice, while the inverted water maze assessed cognitive flexibility in mice, and the experimental steps followed a modified version of a previous experimental protocol [15]; the experiments were carried out in a circular water maze, with a diameter of 1.2 m; the water temperature was maintained at 21–23 °C; some distal tips were posted on the walls around the pool, and the laboratory environment was quiet and undisturbed by the outside world. Each mouse performed three 60 s trials every afternoon for five consecutive days, with 30 min intervals between the trials; the target was an underwater platform located 0.5 cm below the water surface in one of the virtual quadrants (the target quadrant). At the beginning of each trial, a mouse was released into the pool from one of the three virtual quadrants that did not contain the platform, and if the mouse failed to find the platform, the experimenter gently guided it to the platform; the mice were left on the platform for 30 s. The time taken to find the hidden platform was recorded as the escape latency. The platform position remained unchanged over the five training days, and, on Day 6, the mice were tested in regard to their spatial memory by removing the platform, placing the mice in the water, facing a wall opposite the target quadrant. The average swimming speed of the mice, the time required to reach the platform location for the first time, the time spent in each quadrant, the distance traveled, and the number of crossings across the platform area in 60 s were recorded.

The rMWM training began 1 day after the sMWM memory test. The removed platform was put back into the pool, but in the opposite quadrant to the one used during sMWM training. The remote tip on the wall remained in the same position and was in the same position relative to the pool. The mice were given an additional three days to learn the new platform position. The overall process was the same as that described in regard to the sMWM test. On Day 10, the mice underwent a 60 s memory test without a platform. Their movements were tracked using the Noldus EthoVision XT 16 system produced by Noldus Information Technology BV, based in Wageningen, The Netherlands.

### 2.4. Cell Culture and Treatment

The HT22 cell line was obtained from the American Type Culture Collection (ATCC). These cells were maintained in Dulbecco’s Modified Eagle Medium (DMEM, Gibco, Grand Island, NY, USA), supplemented with 10% fetal bovine serum (Gibco) and penicillin–streptomycin (100 U/mL, Gibco). The culture conditions included incubation at 37 °C in a humid atmosphere, containing 5% CO_2_. For the experimental treatments, the cells were exposed to various concentrations of HCY (Sigma, St. Louis, MO, USA) for a duration of 24 h.

### 2.5. VitB Treatment

The RS + VitB group was given 0.2 mL of multivitamin B, daily, through intragastric administration during the MWM tests, and the contents of each component were: VitB_6_ 24 mg/kg/day; VitB_12_ 20 μg/kg/day; and folic acid 10 mg/kg/day. The control and RS group were given the same volume of pure water through intragastric administration [23].

### 2.6. Enzyme-Linked Immunosorbent Assay (ELISA)

The hippocampal tissues and plasma were removed from the mice and prechilled PBS was added. The sample mixture was homogenized using a grinder. After centrifugation at 1000× *g* for 10 min at 4 °C, the hippocampal tissue supernatant was processed using a Mouse HCY ELISA kit (Blue gene, E003H0031, Shanghai, China), following the manufacturer’s instructions [34].

### 2.7. Immunofluorescence (IF) Staining

To assess the immunohistofluorescence, the brains were separated and post-fixed in 4% paraformaldehyde (PFA) at 4 °C overnight. The fixed brains were dehydrated in 10–30% (*w*/*v*) sucrose for cryoprotection and embedded in a Tissue-Tek O.C.T. compound (SAKURA). The brain samples were cut into 25 µm thick sections using a Leica CM1950 cryostat (Leica, Wetzlar, Germany) and subjected to immunohistofluorescence analysis, following routine protocols as described previously. C-fos (1:500, CST, 2250T) was used for staining. The secondary antibodies included Goat anti-Rabbit IgG H&L (Alexa Fluor^®^ 488) (1:200, Abcam, Cambridge, MA, USA, ab150077). The cell nuclei were stained with 4′,6-diamino-2-phenylindole (DAPI) (Sigma–Aldrich, St. Louis, MO, USA). Images were captured using a confocal laser scanning microscope (Olympus, Tokyo, Japan) [34].

### 2.8. Quantitative Real-Time PCR (qRT-PCR)

The total RNA was extracted from HT22 cells and the mouse hippocampus samples using the TRIzol reagent (#93289, Sigma-Aldrich) and were subsequently reverse transcribed into cDNA using the RT Master Mix (#G490, Abmart, Richmond, BC, Canada). A quantitative real-time PCR (qRT-PCR) was performed via the LightCycler 96 Real-time PCR System (Roche, Basel, Switzerland), using the TB Green Pre-mix Ex Taq Kit (TaKaRa, Kyoto, Japan). Moreover, β-Actin was used as an endogenous control. The relative expression levels of the target genes were determined using the 2^-ΔΔCt^ method. The primers for PIN1 (forward 5′-GAAGATGGCGGACGAGGAGAAG-3′, reverse 5′-ACTGGCTGGCGTTGGTGATG-3′) and β-actin (forward 5′-GGCTGTATTCCCCTCCATCG-3′, reverse 5′-CCAGTTGGTAACAATGCCATGT-3′) were used in this study [34].

### 2.9. Western Blotting

The hippocampal tissues and cells were freshly harvested and lysed completely using RIPA buffer, fortified with a protease inhibitor cocktail (MCE, Monmouth Junction, NJ, US). The lysate was centrifuged at 12,000× *g* for 20 min at 4 °C to isolate the supernatant. Subsequently, 25 μg of the protein lysate was loaded onto an 8–12% SDS-PAGE gel. The resolved proteins were transferred onto PVDF membranes, which were then blocked with 5% non-fat dry milk in TBST buffer for 1 h at room temperature. The membranes were incubated with primary antibodies targeting PIN1 (1:1000, Cat# 3722, CST) and β-actin (1:1000, 66009-1-Ig, ProteinTech, Wuhan, China), overnight, at 4 °C. Afterwards, the membranes were incubated with an HRP-conjugated secondary antibody (1:5000, Immunoway, Plano, USA) for 2 h at room temperature. The membranes were treated using an ECL Western blotting substrate kit (MCE, Monmouth Junction, NJ, USA), and the signals were detected using the Image Quant LAS 4000 system (GE, Boston, MA, USA) and were analyzed using ImageJ (RRID: SCR_003070, NIH, USA) software.

### 2.10. Statistical Analysis

The data analysis was performed using GraphPad Prism 9.0, with the results presented as the mean ± SEM. The normality of the continuous variables was assessed. For comparisons between two independent groups, a two-tailed unpaired Student *t*-test was employed. When comparing more than three groups, a one-way ANOVA was conducted, followed by Tukey’s multiple comparisons test. A *p*-value < 0.05 was considered statistically significant.

## 3. Results

### 3.1. Cognitive Flexibility Impairment Induced by Stress

**Figure 1:**
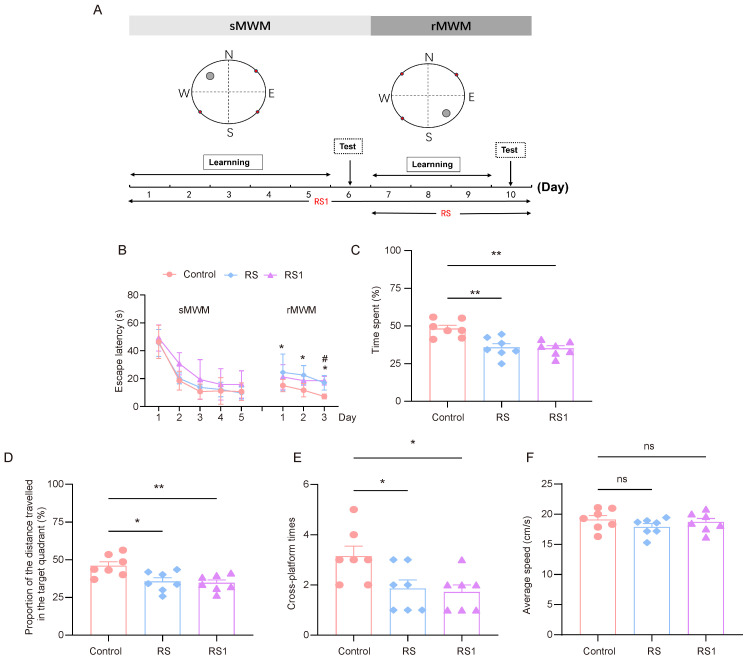
Cognitive flexibility impairment induced by restraint stress. (**A**) Time course of restraint stress and MWM test. (**B**) Changes in escape latency in sMWM and rMWM learning tasks in the control, RS, and RS1 groups (*n* = 7, two-way ANOVA with Tukey’s post hoc test vs. Rs; # *p* < 0.05 vs. RS1 * *p* < 0.05). (**C**) In the rMWM test task, the proportion of time that the mouse spent in the target quadrant compared to the total time, (*n* = 7, one-way ANOVA with Tukey’s post hoc test, ** *p* < 0.01). (**D**) In the rMWM test task, the proportion of distance traveled by the mouse in the target quadrant compared to the total distance traveled (*n* = 7, one-way ANOVA with Tukey’s post hoc test, ** *p* < 0.01, * *p* < 0.05). (**E**) The number of times the platform was crossed in the target quadrant during the rMWM test task (*n* = 7, one-way ANOVA with Tukey’s post hoc test, * *p* < 0.05). (**F**) The average moving speed of the mice during the rMWM test task (*n* = 7, one-way ANOVA with Tukey’s post hoc test, ns *p* > 0.05).

The model of cognitive flexibility impairment was established by using restraint as a stressor. In order to select the appropriate restraint time, the RS group (the restraint began before the rMWM task) and the RS1 group (the restraint began before the sMWM task) were set up. The experimental process is shown in Figure 1A. In regard to the sMWM learning task, it was observed that the time taken to find the escape platform in the three groups of mice was gradually shortened, and the time taken to find the escape platform in the RS1 group was higher than that of the control group and the RS group, but there was no statistically significant difference. In regard to the rMWM learning task, the mice in the RS group spent significantly more time looking for the escape platform than the mice in the control group, and the mice in the RS1 group spent significantly more time looking for the escape platform on the third day only compared to the mice in the control group (Figure 1B).

In regard to the rMWM test task, the proportion of time spent in the target quadrant where the escape platform was located, the proportion of distance traveled in the target quadrant compared to the total distance travelled, and the number of times the hidden platform was crossed were significantly lower in the RS and RS1 groups than in the control group (Figure 1C–E), but there was no significant difference in movement speed among the three groups, which reflected their ability to swim, and the results showed that their ability to move was not impaired. (Figure 1F). These results suggest that restraint stress could lead to impairment of cognitive flexibility in mice.

### 3.2. Stress Leads to a Decrease in C-fos Activation and High PIN1 Expression in the Hippocampus of Mice

**Figure 2:**
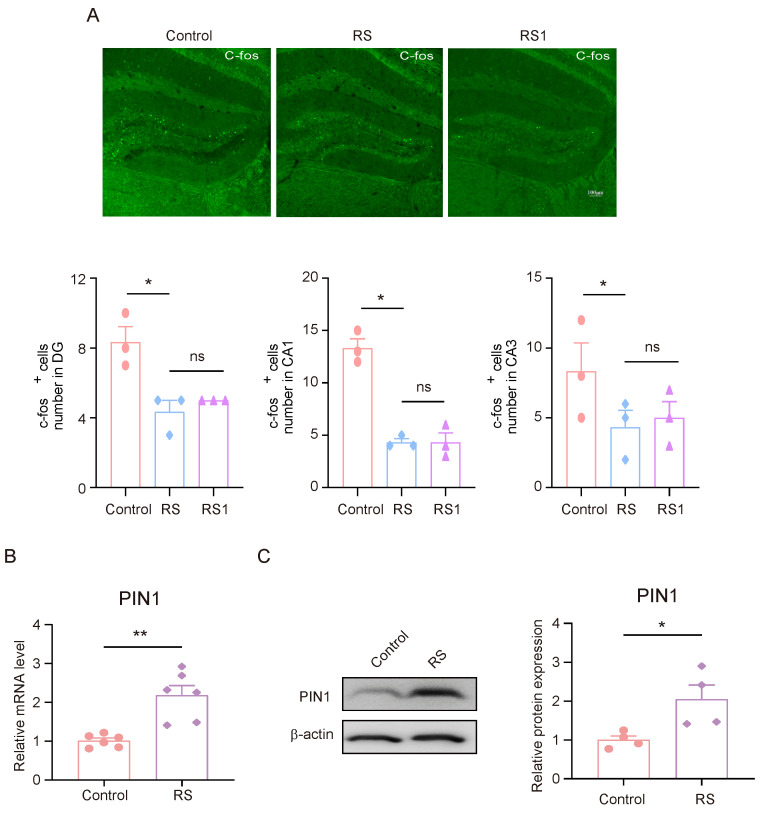
The decrease in C-fos activation and high PIN1 expression in the hippocampus of mice exposed to stress. (**A**) Representative images of C-fos staining in control, RS, and RS1 mice. Scale bar, 50 μm. (* *p* < 0.05, ns: no significance) (**B**) The mRNA expression of PIN1 in hippocampal lysates from mice (*n* = 6, Student’s *t*-test, ** *p* < 0.01). (**C**) The protein expression of PIN1 in hippocampus lysates from mice (*n* = 4, Student’s *t*-test, * *p* < 0.05).

Between 1–1.5 h after the rMWM test task took place, brain tissue samples were collected for the immunofluorescence experiments. The expression of C-fos in DG, CA1, and CA3 in mice was detected, and it was found that the expression of C-fos in DG, CA1, and CA3 in mice in the RS group and the RS1 group was lower than that of the control group, but there was no significant difference between the RS group and the RS1 group (Figure 2A). These results suggest that the hippocampus may play an important role in the development of cognitive flexibility impairment induced by stress. Since the mice in the RS group showed impaired behavioral changes in regard to their cognitive flexibility earlier in the rMWM learning task than that of the RS1 group, and there was an alteration in their C-fos expression, we suggested that the RS group may be a better candidate for the development of cognitive flexibility impairment; in the follow-up experiments, the mice in the RS group were selected as the research objects. Meanwhile, we detected changes in PIN1 in the hippocampus, and the results showed that the mRNA and protein expression levels of PIN1 were increased in the RS group compared with the control group (Figure 2B,C). This suggests that PIN1 may be involved in the impairment of cognitive flexibility induced by restraint stress.

### 3.3. Impairment of Cognitive Flexibility Caused by HCY Participating in Stress

**Figure 3:**
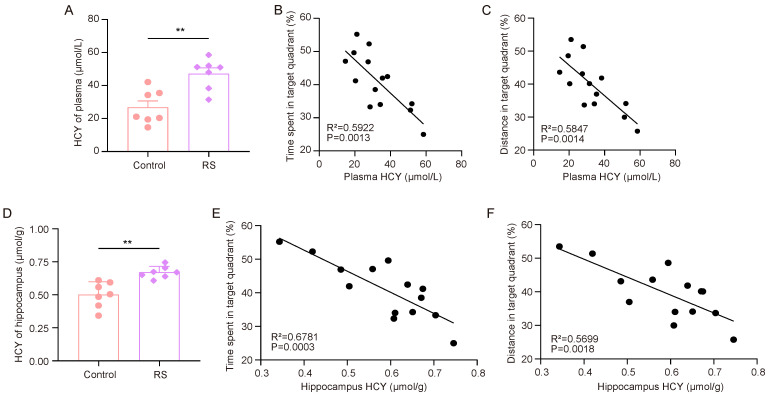
HCY accumulation is associated with stress-induced decline in cognitive flexibility. (**A**) Concentrations of HCY in the plasma of mice (*n* = 7, Student’s *t*-test, ** *p* < 0.01). (**B**,**C**) Correlation analysis of time spent in target quadrant, distance traveled, and plasma HCY in mice during rMWM test task (*n* = 7, simple linear regression). (**D**) Concentration of HCY in the hippocampus of mice (*n* = 7, Student’s *t*-test, ** *p* < 0.01). (**E**,**F**) Correlation analysis of time spent in target quadrant, distance traveled, and HCY in mouse hippocampus during rMWM test task (*n* = 7, simple linear regression).

First, we measured the HCY levels in the plasma and hippocampus of the mice. The ELISA results showed that the HCY levels in the plasma and hippocampus of RS group mice were significantly increased (Figure 3A,D). After that, we analyzed the correlation between the HCY content in mouse plasma and the hippocampus and the proportion of time spent in the target quadrant where the escape platform was located and the proportion of distance traveled in the target quadrant compared to the total distance travelled (Figure 3B,C,E,F). It was found that the performance of the mice in the rMWM test task was negatively correlated with the content of HCY in peripheral plasma and the hippocampus.

### 3.4. HCY Induces Increased PIN1 Expression in Neurons

**Figure 4:**
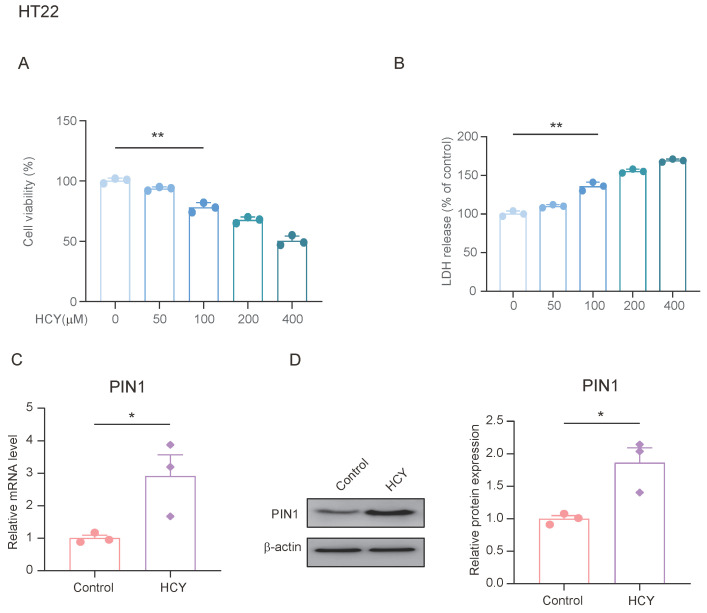
HCY induces an increase in PIN1 expression in HT22 cells. (**A**) HT22 cells were treated with the indicated concentrations of HCY for 24 h, and cell growth was evaluated using a cell counting kit-8 (CCK-8) assay, in regard to three independent experiments. The values are presented as means ± SD (*n* = 3). Statistical analysis was performed using a one-way ANOVA; ** *p* < 0.01. (**B**) HCY induced lactate dehydrogenase (LDH) release in HT22 cells. The values are presented as means ± SD (*n* = 3). Statistical analysis was performed using a one-way ANOVA; ** *p* < 0.01. (**C**) The mRNA expression of PIN1 in HT22 cells (*n* = 3, Student’s *t*-test, * *p* < 0.05). (**D**) The protein expression of PIN1 in HT22 cells (*n* = 3, Student’s *t*-test, * *p* < 0.05).

Previous studies have shown that stress upregulates the expression of PIN1 in the hippocampus of mice, and can lead to an increase in plasma and hippocampal HCY. We treated HT22 cells with different concentrations of HCY for 24 h to determine its effects on cell viability. As shown in Figure 4A, cell viability decreased in a dose-dependent manner after treatment. We also examined the lactate dehydrogenase (LDH) content in the cell supernatants from each group and found that the LDH content in the HCY-treated cells was significantly increased (Figure 4B). To evaluate the effect of HCY on PIN1 expression, we treated HT22 cells with 100 μmol/L of HCY for 24 h and found that HCY significantly upregulated the expression of PIN1 compared with the control group. This suggests that HCY may be involved in the regulation of PIN1 expression.

### 3.5. HCY Increases PIN1 Expression in Mice with Cognitive Impairment

**Figure 5:**
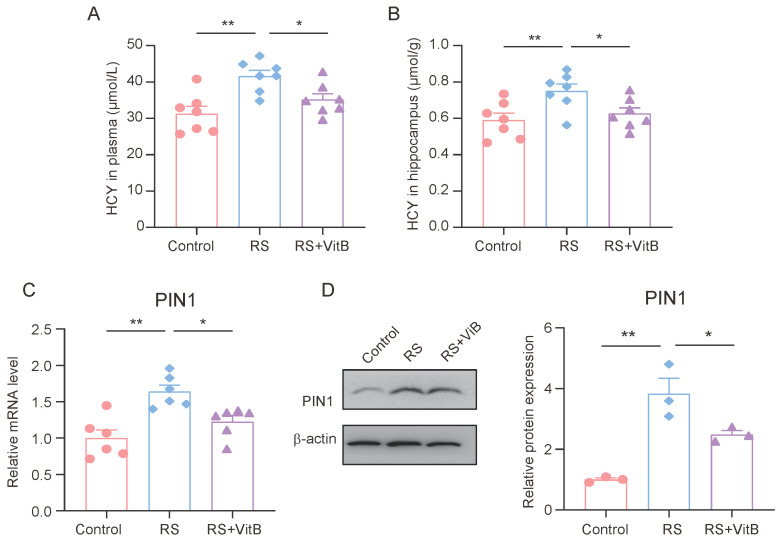
HCY increases PIN1 expression in mice with cognitive impairment. (**A**) Concentrations of HCY in the plasma of mice (*n* = 7, one-way ANOVA with Tukey’s post hoc test, ** *p* < 0.01, * *p* < 0.05). (**B**) Concentrations of HCY in the hippocampus of mice (*n* = 7, one-way ANOVA with Tukey’s post hoc test, ** *p* < 0.01, * *p* < 0.05). (**C**) The mRNA expression of PIN1 in hippocampus lysates (*n* = 6, one-way ANOVA with Tukey’s post hoc test, ** *p* < 0.01, * *p* < 0.05). (**D**) The protein expression of PIN1 in hippocampus lysates (*n* = 3, one-way ANOVA with Tukey’s post hoc test, ** *p* < 0.01, * *p* < 0.05).

At the same time, we detected the HCY content in the plasma and hippocampus of the mice. The ELISA results showed that VitB could ameliorate the increase in HCY in plasma and the hippocampus induced by restraint stress in mice (Figure 5A,B). In addition, we also detected the expression of PIN1 in the hippocampus of mice. It was found that VitB could also inhibit the high expression of PIN1 in the hippocampus of mice induced by restraint stress (Figure 5C,D).

### 3.6. Regulation of HCY Ameliorates Stress-Induced Impairment of Cognitive Flexibility

**Figure 6:**
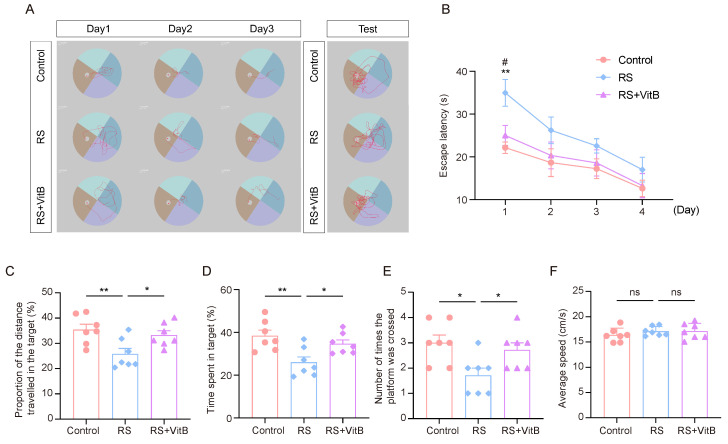
VitB ameliorates cognitive flexibility impairment induced by restraint stress by regulating HCY. (**A**) Representative trajectory plots of mice in regard to the rMWM learning and testing task. (**B**) Escape latency in regard to rMWM test (*n* = 7, one-way ANOVA with Tukey’s post hoc test, RS + VitB vs. RS, # *p* < 0.05, control vs. RS, ** *p* < 0.01). (**C**) In the rMWM test task, the proportion of time that the mice spent in the target quadrant compared to the total time (*n* = 7, one-way ANOVA with Tukey’s post hoc test, ** *p* < 0.01, * *p* < 0.05). (**D**) In the rMWM test task, the proportion of the distance traveled by the mouse in the target quadrant compared to the total distance traveled (*n* = 7, one-way ANOVA with Tukey’s post hoc test, ** *p* < 0.01, * *p* < 0.05). (**E**) The number of times the mice crossed the platform in the target quadrant during the rMWM test task (*n* = 7, one-way ANOVA with Tukey’s post hoc test, * *p* < 0.05). (**F**) The average moving speed of the mice in the rMWM test task (*n* = 7, one-way ANOVA with Tukey’s post hoc test).

We used VitB to interfere with HCY in mice and performed the MWM tests (Figure 6A). It was found that the RS + VitB group spent less time looking for the escape platform in the rMWM learning task than the RS group, and the difference was most significant on the first day (Figure 6B). In regard to the rMWM test task, the proportion of time spent in the target quadrant where the escape platform was located, the proportion of distance traveled in the target quadrant compared to the total distance travelled, and the number of times the hidden platform was crossed were significantly higher in the RS + VitB group than in the RS group, but no significant difference in swimming speed was observed (Figure 6C–F). These results suggest that reducing HCY in mice can reverse the impairment of cognitive flexibility induced by restraint stress.

## 4. Discussion

The impairment of cognitive flexibility due to stress has been confirmed by many studies. Acute stress impairs prefrontal cortex function, which in turn reduces task switching. Shields et al. found in a psychological stress-induced experiment that the stressed group made significantly more consistent errors in the Wisconsin card sorting task than the control group, indicating impaired cognitive flexibility [35,36,37]. In addition, chronic stress can also have a negative impact on cognitive flexibility, especially in regard to a long-term stress environment; individual creativity and adaptive thinking ability will be significantly reduced. Hippocampal neurons showed obvious damage under stress conditions. Chronic stress impairs long-term potentiation (LTP) in the hippocampal CA1 region, which in turn affects learning and memory [38,39]. In addition, high concentrations of cortisol can damage hippocampal neurons through a variety of mechanisms, including inhibiting mitochondrial function, increasing oxidative stress, downregulating Neurotrophin (such as BDNF) expression, and activating apoptosis pathways. These changes eventually lead to atrophy or apoptosis of hippocampal neurons, which in turn affects cognitive flexibility [40,41,42].

HCY is a sulfur-containing amino acid, and its metabolic abnormalities are associated with a variety of neurological disorders [28,43]. Previous studies in the laboratory have found that stress leads to abnormally high HCY in mice, and participates in their cognitive function and emotional abnormality [10]. PIN1 is an enzyme that regulates protein phosphorylation and plays an important role in the regulation of nervous system function. PIN1’s role in regulating neuronal excitability and synaptic plasticity makes it a key regulator of stress-induced impairments in cognitive flexibility. Elevated PIN1 expression further affects the antioxidant capacity of neurons, exacerbates oxidative damage, and ultimately leads to impaired cognitive flexibility [44,45,46]. Our results show that PIN1 function is suppressed in the context of stress-induced cognitive flexibility impairment, further exacerbating cognitive flexibility impairment. HCY may be indirectly involved in the regulation of cognitive flexibility by affecting the expression of PIN1.

Cognitive flexibility extends throughout a person’s life. Aging, disease, and the environment may lead to impaired cognitive flexibility and affect an individual’s quality of life. Therefore, the prevention and treatment of cognitive flexibility impairment is particularly important. In AD, PD, and amyotrophic lateral sclerosis (ALS), decreased cognitive flexibility is strongly associated with impaired white matter fiber integrity, dysfunction of the executive control network, and dysfunction of the salience network. In obsessive–compulsive disorder, reduced cognitive flexibility is thought to be linked to dysfunctional neural circuits between the cortex and the basal ganglia. Studies have shown that people with OCD show significant abnormalities in neural activity during tasks, particularly in the prefrontal and striatum regions [47,48,49]. However, the mechanism of and prevention strategies for the impairment of cognitive flexibility are not fully understood.

Therefore, this study explored the relationship between impairment of cognitive flexibility and an abnormal increase in HCY during restraint stress from the perspective of HCY metabolism. Our results show that restraint stress can lead to elevated expression of PIN1 in the hippocampus of mice, in addition to abnormally elevated plasma and hippocampal HCY levels in mice. VitB, as a metabolic regulator of HCY, was found to alleviate the increase in hippocampal PIN1 and HCY induced by restraint stress in mice, which in turn reversed the decline of cognitive flexibility induced by restraint stress in mice.

## 5. Conclusions

In conclusion, the present study provides evidence that restraint stress can lead to impairment of cognitive flexibility in mice, with elevated PIN1 expression in the hippocampus of mice, accompanied by elevated HCY content in the plasma and hippocampus of mice. VitB can alleviate the impairment of cognitive flexibility and PIN1 expression caused by restraint stress by regulating HCY metabolism. Our current findings connect HCY with the decline of cognitive flexibility caused by restraint stress, and provide a new perspective and evidence for further exploration and research on the mechanism of cognitive flexibility impairment.

## Data Availability

The datasets generated during and/or analyzed during the current study are available from the corresponding author on reasonable request. The data are not publicly available due to specific ethical and privacy considerations.

## Abbreviations

The following abbreviations are used in this manuscript:

PIN1	Peptidyl-prolyl cis-trans isomerase NIMA-interacting 1
AD	Alzheimer’s disease
PD	Parkinson’s disease
HCY	Homocysteine
BBB	Blood–brain barrier
ALS	Amyotrophic lateral sclerosis
OCD	Obsessive–compulsive disorder
